# *Burkholderia pseudomallei* BimC Is Required for Actin-Based Motility, Intracellular Survival, and Virulence

**DOI:** 10.3389/fcimb.2019.00063

**Published:** 2019-03-22

**Authors:** Varintip Srinon, Somjit Chaiwattanarungruengpaisan, Sunee Korbsrisate, Joanne M. Stevens

**Affiliations:** ^1^Department of Immunology, Faculty of Medicine Siriraj Hospital, Mahidol University, Bangkok, Thailand; ^2^Microbiology Laboratory, Faculty of Veterinary Science, Veterinary Diagnostic Center, Mahidol University, Nakhon Pathom, Thailand; ^3^The Monitoring Surveillance Center for Zoonotic Diseases in Wildlife and Exotic Animals, Faculty of Veterinary Science, Mahidol University, Nakhon Pathom, Thailand; ^4^The Roslin Institute and Royal (Dick) School of Veterinary Studies, University of Edinburgh, Midlothian, United Kingdom

**Keywords:** *Burkholderia pseudomallei*, BimA, BimC, actin-based motility, intracellular survival, multi-nucleated giant cell, virulence

## Abstract

The intracellular pathogen *Burkholderia pseudomallei*, the etiological agent of melioidosis in humans and various animals, is capable of survival and movement within the cytoplasm of host cells by a process known as actin-based motility. The bacterial factor BimA is required for actin-based motility through its direct interaction with actin, and by mediating actin polymerization at a single pole of the bacterium to promote movement both within and between cells. However, little is known about the other bacterial proteins required for this process. Here, we have investigated the role of the *bimC* gene (*bpss1491*) which lies immediately upstream of the *bimA* gene (*bpss1492*) on the *B. pseudomallei* chromosome 2. Conserved amongst all *B. pseudomallei, B. mallei* and *B. thailandensis* strains sequenced to date, this gene encodes an iron-binding protein with homology to a group of proteins known as the bacterial autotransporter heptosyltransferase (BAHT) family. We have constructed a *B. pseudomallei bimC* deletion mutant and demonstrate that it is defective in intracellular survival in HeLa cells, but not in J774.1 macrophage-like cells. The *bimC* mutant is defective in cell to cell spread as demonstrated by ablation of plaque formation in HeLa cells, and by the inability to form multi-nucleated giant cells in J774.1 cells. These phenotypes in intracellular survival and cell to cell spread are not due to the loss of expression and polar localization of the BimA protein on the surface of intracellular bacteria, however they do correlate with an inability of the bacteria to recruit and polymerize actin. Furthermore, we also establish a role for *bimC* in virulence of *B. pseudomallei* using a *Galleria mellonella* larvae model of infection. Taken together, our findings indicate that *B. pseudomallei* BimC plays an important role in intracellular behavior and virulence of this emerging pathogen.

## Introduction

*Burkholderia pseudomallei*, a Gram-negative environmental saprophyte, is the causative agent of melioidosis, a potentially life-threatening infectious disease affecting both humans and animals in certain areas of the tropics (Cheng and Currie, 2005). The pathogen is endemic in South East Asia (Limmathurotsakul et al., 2014) and Northern Australia where the bacterium has been detected in drinking water and soil (Currie et al., 2001; Kaestli et al., 2009; Draper et al., 2010). The infection is typically acquired through inhalation, skin penetration or possibly through ingestion; and the resulting disease can manifest with clinically diverse signs including skin abscesses, severe pneumonia and septic shock (Cheng et al., 2013). In North East Thailand, the case fatality rate of melioidosis is around 40% and the incidence of melioidosis continues to increase annually (Limmathurotsakul et al., 2010). Additionally, recent modeling data suggests that the incidence of melioidosis is around 165,000 cases per year worldwide, of which 89,000 are fatal (Limmathurotsakul et al., 2016). *B. pseudomallei* has gained much attention in recent years over concerns regarding its potential for use as a biological warfare agent. The aerosol infectivity, high mortality rate, and the absence of an effective human vaccine for the prevention of melioidosis has resulted in *B. pseudomallei* being listed as a Tier 1 select agent in the U.S. There is a continuing need to characterize virulence mechanisms of *B. pseudomallei* pathogenesis to inform novel strategies for disease prevention and control.

As a facultative intracellular bacterium, *B. pseudomallei* is capable of entering both phagocytic and non-phagocytic cells. Following phagocytosis, *B. pseudomallei* has evolved mechanisms to escape the phagosome into the host cell cytoplasm (Jones et al., 1996; Harley et al., 1998; Kespichayawattana et al., 2000), where it replicates and causes host cell death by induction of apoptosis (Kespichayawattana et al., 2000; Sun et al., 2005). Whilst in the cytoplasm, *B. pseudomallei* subverts host cellular actin dynamics to induce actin polymerization at one bacterial pole leading to its movement within and between infected cells by a process known as actin-based motility, as seen for several other intracellular pathogens (Stevens et al., 2002, 2005b; Gouin et al., 2005). Actin-based motility is thought to underlie the ability of *B. pseudomallei* to spread into adjacent cells in the absence of immune surveillance, thereby contributing to the formation of multi-nucleated giant cells (MNGCs) (Kespichayawattana et al., 2000) and virulence. This unique phenotype may be relevant to pathogenesis, since granuloma formation and generation of MNGCs are also found in tissues of melioidosis patients (Wong et al., 1995). Infection of J774.2 murine macrophage-like cells with a *B. pseudomallei bimA* mutant showed a complete lack of actin tail formation, demonstrating that this process is dependent on the BimA protein (Stevens et al., 2005b), a putative Type V autosecreted protein exhibiting carboxyl-terminal homology to the oligomeric *Yersinia* adhesion protein and bacterial autotransporter YadA (Stevens et al., 2005b; Lazar Adler et al., 2011). The BimA protein is encoded by all *B. pseudomallei* strains sequenced to date (Sitthidet et al., 2008). BimA is anchored to one pole of the bacterial outer membrane during actin tail formation in infected cells, interacts directly with cellular actin and is capable of polymerizing actin into filaments in the absence of any other cellular or bacterial proteins (Stevens et al., 2005b). Specific domains of the BimA protein with homology to eukaryotic proteins have been implicated in actin polymerization and cell to cell spread (Sitthidet et al., 2011).

Recently a proteomic approach has identified candidate host cell proteins involved in actin polymerization mediated by BimA (Jitprasutwit et al., 2016). For example the cellular scaffold protein IQGAP1 plays a role in regulating the actin density and tail length of *B. pseudomallei* in infected HeLa cells (Jitprasutwit et al., 2016). Interestingly, BimA proteins from the closely related virulent *B. mallei* and the avirulent *B. thailandensis* differ significantly in amino acid sequence at the N-terminus of the proteins, resulting in the use of distinctly different mechanisms of actin polymerization between *B. pseudomallei* and *B. thailandensis* (Stevens et al., 2005a; Sitthidet et al., 2010; Benanti et al., 2015).

The *B. pseudomallei bimA* gene (annotated as *bpss1492* in the reference K96243 genome) is encoded on the second smaller chromosome within an operon of several co-regulated genes, including genes encoding the VirAG two component system required for regulation of the virulence-associated T6SS of *B. pseudomallei* (Burtnick and Brett, 2013). *B. pseudomallei bimA* lies downstream of a gene encoding a putative glycosyltransferase (*bpss1491, bimC*). The predicted BimC protein shows amino acid sequence homology with members of the bacterial autotransporter heptosyltransferase (BAHT) family (Lu et al., 2014) including the diarrheagenic *Escherichia coli* AAH protein (Autotransporter Adhesin Heptosyltransferase). AAH protein catalyzes the addition of heptose molecules to the *Escherichia coli* virulence factor AIDA-I (Adhesin Involved in Diffuse Adherence) to mediate bacterial autoaggregation and adhesion to the host cell (Sherlock et al., 2004; Benz and Schmidt, 2011). Interestingly, a *Tn*5-based transposon mutagenesis screen in *B. thailandensis* revealed a role for the *bimC* gene in actin tail formation and MNGC formation (Lu et al., 2015). In addition, the authors demonstrated that expression of an N-terminal tagged *B. thailandensis* BimC protein from a prokaryotic vector was required for polar localization of a similarly expressed N-terminal tagged *B. thailandensis* BimA protein, likely facilitated through a direct interaction between the two proteins (Lu et al., 2015).

In the present study, we aimed to characterize the role of native BimC protein in a virulent *B. pseudomallei* strain. We constructed and characterized a *B. pseudomallei* Δ*bimC* deletion mutant and determined its role in *B. pseudomallei* intracellular survival and virulence. Interestingly we showed that the mutant was defective in net intracellular replication in HeLa cells, a finding that was not reproduced in the macrophage-like cell line J774.1. Unlike the findings of Lu et al. (2015), who studied the *B. thailandensis bimC* gene, deletion of *bimC* in *B. pseudomallei* did not affect the expression or polar localization of native BimA protein on the surface of intracellular bacteria, although it did prevent actin recruitment and polymerization. The ability of the *B. pseudomallei* Δ*bimC* deletion mutant to induce plaques and MNGCs were also studied *in vitro*, and the role of *bimC* in pathogenesis was assessed using a *Galleria mellonella* larvae surrogate model of infection.

## Materials and Methods

### Bacterial Strains, Cell Lines, and Culture Conditions

*B. pseudomallei* strain 10276, a clinical isolate from a human melioidosis patient, was kindly provided by Prof. Ty Pitt (Health Protection Agency, Colindale, United Kingdom) (Maegraith and Leithead, 1964). Construction of the Δ*bimA* mutant is described in Jitprasutwit et al. (2016). All experiments with *B. pseudomallei* and its derivatives were conducted in a Biosafety Level-3 Laboratory at Mahidol University with Institutional Biosafety Committee approval. *E. coli* DH5α, λ*pir*-116, and S17-1 λ*pir* strains were used as bacterial host strains for molecular cloning or conjugation. Bacteria were routinely cultured at 37°C in Luria-Bertani (LB; Difco) agar or broth. Chloramphenicol (10 μg/ml for *E. coli* and 50 μg/ml for *B. pseudomallei*) was added to the medium when required. Yeast extract tryptone (YT) agar (Difco) lacking NaCl and supplemented with 15% sucrose was used for resolution of merodiploids during allelic exchange (Logue et al., 2009). Murine macrophage J774A.1 cells and human epithelial HeLa cells were obtained from the American Type Culture Collection (ATCC) and were cultured in Dulbecco's Modified Eagle medium (DMEM; Gibco-BRL) supplemented with 10% heat-inactivated fetal bovine serum (FBS; HyClone) at 37°C in the presence of 5% CO_2_. Unless otherwise stated, all reagents were purchased from Sigma-Aldrich.

### Construction of an Isogenic Δ*bimC* Deletion Mutant and Complemented Strain

Mutagenesis of the *B. pseudomallei bimC* gene was performed using a homology recombination and *sacB* counter-selection approach using the suicide replicon pDM4, essentially as previously described (Logue et al., 2009). To produce the Δ*bimC* deletion mutant, ~400-bp DNA upstream and downstream of *B. pseudomallei bimC* (*bpss1491*) were amplified (BimC-P1/P2 and BimC-P3/P4; Table S1), kinase-treated and ligated before a second round of PCR using primers BimC-P1 and BimC-P4. The resulting PCR product was digested with *Spe*I and *Xba*I before ligating with similarly digested pDM4. The recombinant pDM4 Δ*bimC* plasmid was introduced into *B. pseudomallei* strain 10276 by conjugation. Merodiploids were selected on agar plates containing 50 μg/ml chloramphenicol, and merodiploid resolution was selected by plating on YT agar (without NaCl) supplemented with 15% sucrose at 30°C (Logue et al., 2009). Construction of the Δ*bimC* deletion mutant was confirmed by PCR (using the primers listed in Table S1) and DNA sequencing.

For complementation of the Δ*bimC* mutant, pBHR1-*bimC* encoding the complete *bimC* ORF was constructed by PCR amplification of the 10276 *bimC* gene using the primers listed in Table S1. Following verification by DNA sequencing, the plasmid was introduced into the Δ*bimC* mutant by electroporation (Choi and Schweizer, 2005) to produce the strain Δ*bimC*/pBHR1-*bimC*, which was verified by plasmid DNA extraction and further DNA sequencing.

### Net Intracellular Replication Assay

The net intracellular replication of *B. pseudomallei* in macrophage-like and epithelial cells were assessed as described previously (Muangsombut et al., 2008) with some modifications. Essentially, J774A.1 or HeLa cells were seeded at a density of 2.5 x 10^5^ cells per well of a 24-well tissue culture plate and infected ~24 h later with *B. pseudomallei* wild-type (10276) or its derivatives at a multiplicity of infection (MOI) of 0.5 or 50, respectively. At 2 h post infection, infected cells were overlaid with DMEM medium containing kanamycin (250 μg/ml) to kill extracellular bacteria. The infected cells were subsequently lysed at 2, 6, 12, 18, and 24 h post infection with PBS containing 0.1% Triton X-100. The intracellular bacteria were serially diluted and plated on tryptic soy agar. Colony forming units (CFU) were counted after 36–48 h incubation at 37°C.

### Plaque and MNGC Formation Assays

*Burkholderia*-induced plaque assays were performed as described earlier (Pumirat et al., 2014) with some modifications. Cells were infected with *B. pseudomallei* wild-type or its derivatives at MOI of 50 for 2 h. Then, the infected cell monolayers were overlaid with fresh medium containing 250 μg/ml of kanamycin to kill extracellular bacteria and incubated statically for 24–48 h before crystal violet staining. Plaque-forming efficiency was calculated as the number of plaques divided by bacterial CFU added per well. Detection of *B. pseudomallei*-induced MNGC formation in J774A.1 and HeLa cells was assessed by confocal microscopy.

### Immunostaining and Confocal Microscopy

J774A.1 or HeLa cells were infected with *B. pseudomallei* wild-type or its derivatives at a multiplicity of infection (MOI) of 0.5 or 50, respectively. At the experimental end-point, the infected cells were fixed with 4% (w/v) paraformaldehyde in phosphate-buffered saline (PBS) overnight. Cells were permeabilized with PBS containing 0.1% (v/v) Triton X-100 for 30 min and incubated with PBS containing 1% (w/v) bovine serum albumin (BSA) for 30 min at room temperature. Bacteria were stained with rabbit polyclonal anti-*B. pseudomallei* lipopolysaccharide antibody (kindly provided by Prof. R.W. Titball, Exeter University, UK) at 37°C for 1 h, washed with PBS and then incubated with goat anti-rabbit antibody-Alexa Fluor^488^ (Molecular Probes). Nuclei were stained with DAPI (4', 6-Diamidino-2-Phenylindole, Dihydrochloride; Molecular Probes). F-actin was directly stained using phalloidin^568^ (Molecular Probes). BimA protein was detected with a panel of three previously described monoclonal antibodies (Stevens et al., 2005b). Bound anti-BimA was detected with goat anti-mouse antibody-AlexaFluor^568^ (Molecular Probes). Images were captured using an LSM 510 laser scanning confocal microscope (Carl Zeiss).

### Galleria Mellonella Killing Assay

*G. mellonella* killing assays were performed according to Wand et al. (2011), with some modifications. Larvae between 2 and 2.5 cm and free of melanization or injury were used in the experiments. To prepare the bacterial culture for infection, the overnight culture of *B. pseudomallei* wild-type or its derivatives were adjusted to the concentration to 10^4^ CFU per ml in PBS. A Hamilton syringe was used to inject 10 μl bacterial suspension into the *G. mellonella* larvae. Injections were performed directly into the larval body cavity and groups of 10 larvae were injected with each bacterial strain. Control larvae were injected with PBS. Following injection, larvae were incubated in the dark at 37°C and the number of dead larvae were recorded at a variety of times post injection. The virulence test was performed three times on separate occasions.

### Statistical Analysis

For *in vivo* mutant characterization, a log-rank (Mantel-Cox) test was used to compare survival curves using Graph Pad Prism7 software. Data from the experiments for comparison between groups were collected from three independent experiments and analyzed using the student's unpaired *t*-test using Graph Pad Prism7 software. The results were considered significant if the *P* < 0.05.

## Results

### Generation of a *B. pseudomallei BimC* Deletion Mutant and a Complemented Strain

BimC is completely conserved amongst all *B. pseudomallei, B. mallei, B. thailandensis* and *B. oklahomensis* strains studied to date, with over 90% amino acid identity. Although it is notable that *B. pseudomallei* BimC proteins possess a 13 amino acid C-terminal extension missing from the BimC proteins of the other closely related *Burkholderia* species (Figure S1).

The *bimC* gene (*bpss1491*) is situated directly upstream of the *bimA* gene (*bpss1492*) within an operon of genes including the two-component regulator *virAG* (Figure S2A). To investigate the function of the *bimC* gene, we created a *B. pseudomallei bimC* deletion mutant (Δ*bimC*) using the suicide replicon pDM4 and unmarked allelic-exchange mutagenesis as previously described (Logue et al., 2009). The *bimC* merodiploid strain yielded DNA fragments consistent with both the presence of the wild-type allele and the deletion allele introduced on the pDM4 plasmid (Figure S2B). Following growth on sucrose plates, deletion of the *bimC* gene was confirmed by PCR using primers flanking the deletion region (Figure S2B). This strain was also verified across the deletion junction by DNA sequencing. A constitutive prokaryotic expression plasmid containing *bimC* (pBHR1-*bimC*) was introduced into the Δ*bimC* mutant to give rise to the complemented strain Δ*bimC*/pBHR1-*bimC*. Neither deletion of the *bimC* gene nor introduction of the plasmid-borne *bimC* gene resulted in any detectable difference in growth of the mutants in LB broth compared to the isogenic parent strain 10276 (data not shown).

### BimC Is Essential for Actin-Based Motility of *B. pseudomallei* in Cultured Cells

**Figure 1:**
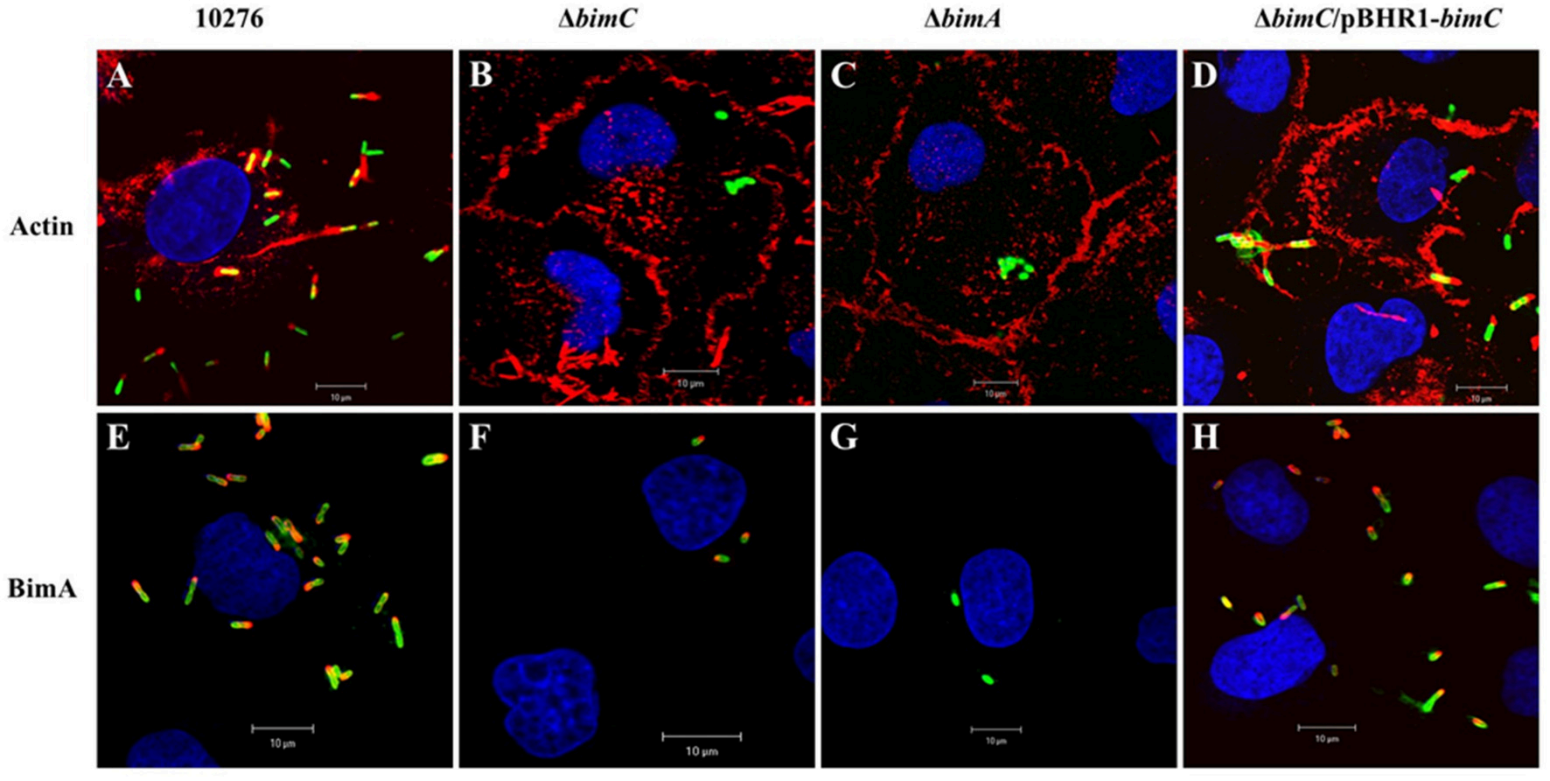
Actin-based motility of *B. pseudomallei* strains in HeLa cells. *B. pseudomallei* wild-type strain 10276 **(A,E)**, Δ*bimC* mutant **(B,F)**, Δ*bimA* mutant **(C,G)**, and Δ*bimC*/pBHR1-*bimC* **(D,H)** strains were used to infect HeLa cells. At 12 h post infection, the infected epithelial cells and bacteria were stained for bacteria and actin-tails **(A–D)**, or bacteria and BimA protein expression **(E–H)**. Bacteria (green) were stained with anti-*B. pseudomallei* lipopolysaccharide antibody and detected with anti-rabbit antibody-AlexaFluor^488^. F-actin (red) was stained with phalloidin-AlexaFluor^568^ and nuclei (blue) were stained with DAPI. BimA protein (red) was stained with a panel of three monoclonal antibodies detected with anti-mouse antibody-AlexaFluor^568^. Scale bar = 10 μm.

*B. pseudomallei* expresses the BimA protein to recruit cellular actin at the pole of the bacterium where it catalyzes its polymerization to promote its movement within and between infected host cells (Stevens et al., 2005b; Sitthidet et al., 2011). To determine the effect of *bimC* deletion on *B. pseudomallei* induced-actin tail formation, HeLa cells were infected with the *B. pseudomallei* 10276 and mutant (Δ*bimC*, Δ*bimA*, and Δ*bimC*/pBHR1-*bimC*) strains, and the cells were analyzed by immunofluorescence staining and confocal microscopy. At 12 h post infection, wild-type *B. pseudomallei* exhibited many intracellular bacteria with intense filamentous actin staining proximal to one bacterial pole (Figure 1A). In contrast, no such membrane protrusions or F-actin filaments were detected in any of the cells infected with the Δ*bimC* or Δ*bimA* mutant (Figures 1B,C), despite demonstrating efficient escape of these strains into the cell cytoplasm that was indistinguishable from WT bacteria (see Figure S3 and Supplemental Methods). The deficiency of actin tail formation of the Δ*bimC* mutant could be partially restored by plasmid-borne *bimC* (Δ*bimC*/pBHR1-*bimC)* (Figure 1D).

Interestingly, at the same time point (12 h post infection), intracellular bacteria with polar expression of the BimA protein were visible in cells infected with the Δ*bimC* mutant, similar to the wild-type strain (Figures 1E,F,H). As expected, the Δ*bimA* mutant lacked any BimA expression (Figure 1G). Similar data was obtained when J774.1 cells were infected with the same *B. pseudomallei* strains (Figure S4). Notably, the Δ*bimC* mutant failed to display actin-based motility, even though intracellular bacteria with polar expression of the BimA protein were detected in infected J774A.1 cells (Figure S4). These results indicate that BimC is required for BimA-mediated *B. pseudomallei* actin-based motility in infected cells, and that unlike the studies on *B. thailandensis bimC* (Lu et al., 2015), *B. pseudomallei bimC* is not required for localization of *B. pseudomallei* BimA at the pole of the bacterium.

### BimC Is Necessary for *B. pseudomallei* Survival in HeLa Cells

**Figure 2:**
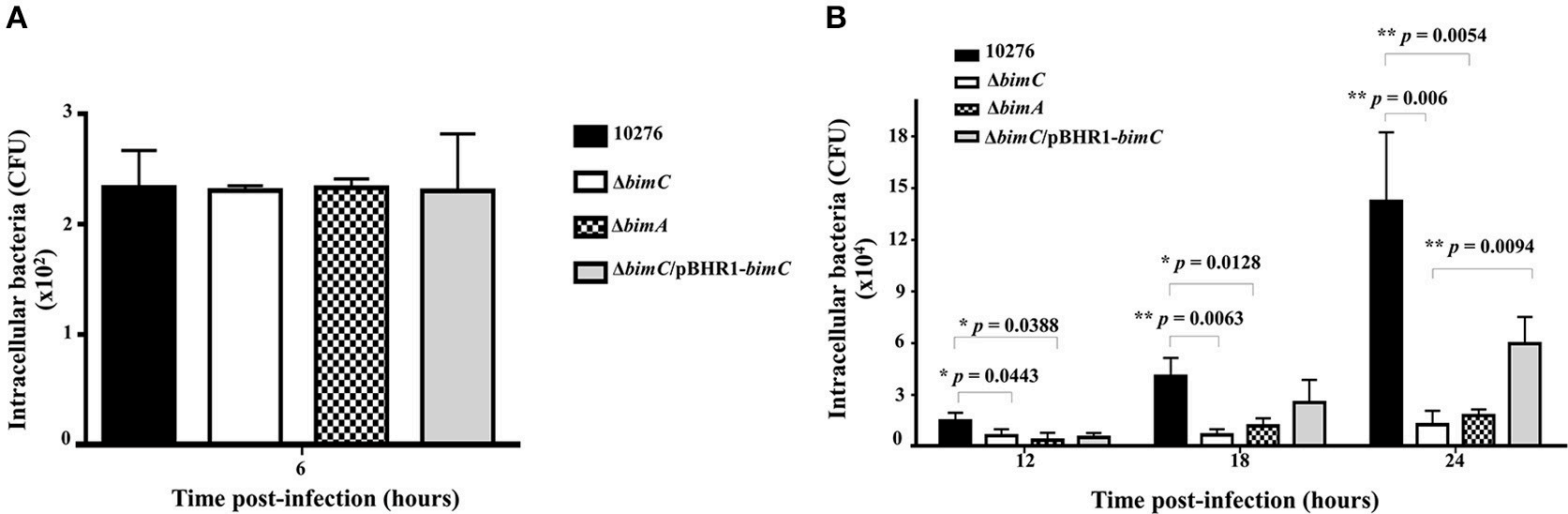
Net intracellular replication and survival of *B. pseudomallei* strains within HeLa cells. HeLa cells were infected with *B. pseudomallei* strains at an MOI of 50. At the indicated time point, the numbers of viable intracellular bacteria (CFU) were determined. Data from 6 h post-infection is shown in **A** and data from 12, 18, and 24 h post-infection in **B**. The graphs show data for the wild-type (black bars), Δ*bimC* mutant (white bars), Δ*bimA* mutant (dotted bars), and Δ*bimC*/pBHR1-*bimC* (gray bars) strains. Error bars represent standard errors of the means from three independent experiments (*n* = 3 biological replicates). Asterisks indicate significant differences (*P* < 0.01, *t*-test).

*B. pseudomallei* has the ability to multiply and survive within a wide range of both phagocytic and non-phagocytic cells. To assess the role of *bimC* in intracellular survival in non-phagocytic cells, the net intracellular replication of the *B. pseudomallei* Δ*bimC* mutant was assessed in comparison with wild-type, Δ*bimC*/pBHR1-*bimC* and Δ*bimA* mutant strains. At 6 h post infection, all *B. pseudomallei* strains showed a similar number of viable bacteria (CFU) within infected cells, without any statistical difference (Figure 2). In contrast, the number of viable Δ*bimC* mutant recovered was significantly lower than the wild-type strain at 12, 18, and 24 h post infection (*P* = 0.0054 at 24 h post infection) (Figure 2B). This defect in intracellular survival and replication could be partially restored by a plasmid-borne *bimC* gene (Figure 2), indicating that *bimC* is required for *B. pseudomallei* intracellular replication within HeLa cells. The intracellular replication defect of the Δ*bimC* mutant is indistinguishable from that of the Δ*bimA* mutant (this study and Sitthidet et al., 2011).

In addition to HeLa cells, intracellular replication within J774A.1 macrophage-like phagocytic cells was also investigated. Interestingly there was no significant difference in the intracellular bacterial load of the wild-type, Δ*bimC*, Δ*bimC*/pBHR1-*bimC*, and Δ*bimA* mutants in J774A.1 cells, across all time points studied (Figure S5). This may highlight a different role for actin-based motility in these two different cell types, or differences in the pathogen recognition and innate immunity pathways these cell types may deploy to control intracellular replication of *B. pseudomallei*.

### BimC Facilitates *B. pseudomallei* Intercellular Spreading

**Figure 3:**
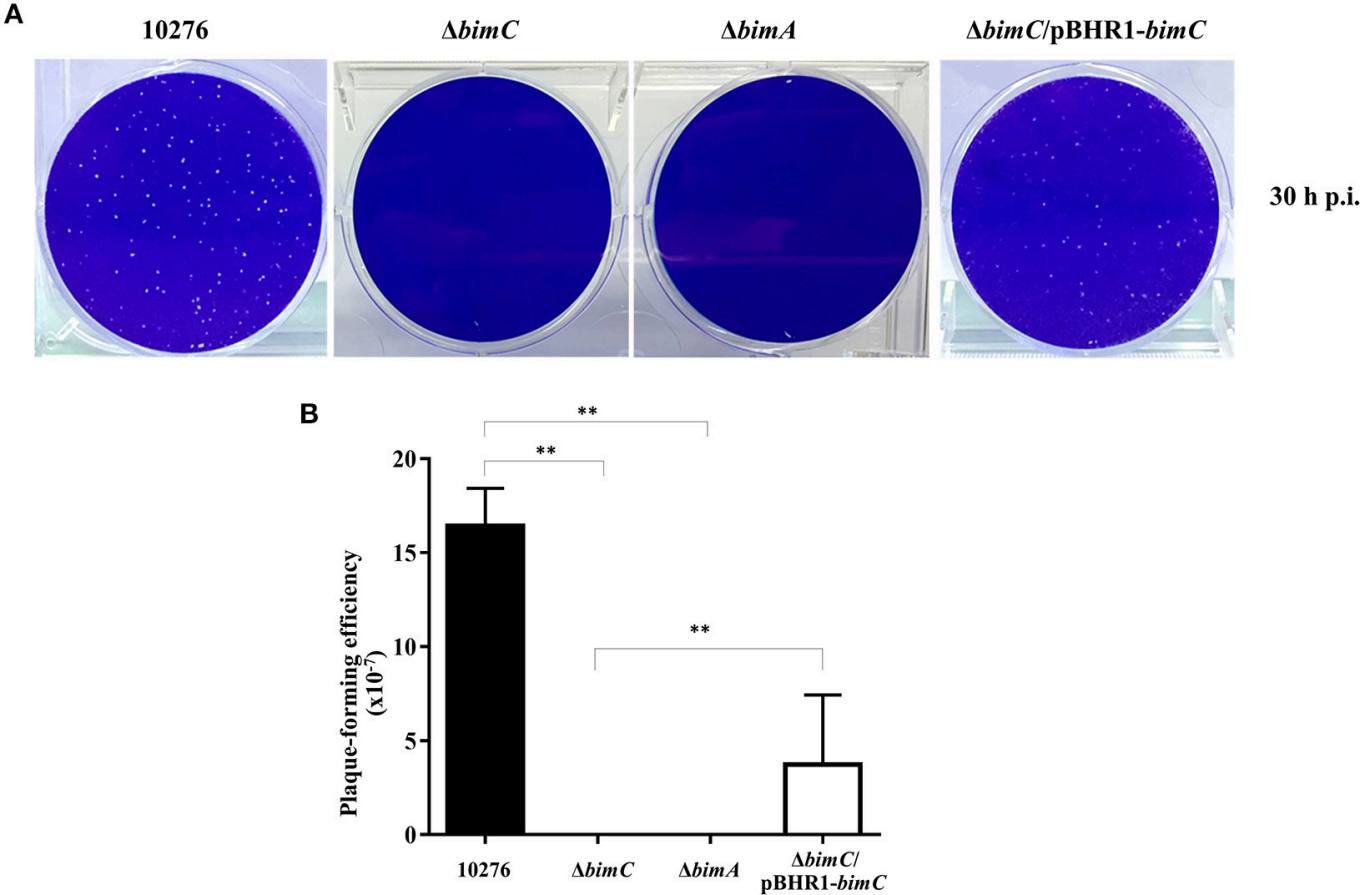
Intercellular spreading of *B. pseudomallei* strains in HeLa cells. **(A)** Representative micrographs of cell monolayers and **(B)** plaque-forming efficiency of HeLa cells infected with *B. pseudomallei* wild-type (10276), Δ*bimC* mutant, Δ*bimA* mutant, or Δ*bimC*/pBHR1-*bimC* strains. Plaque-forming efficiency was calculated as the number of plaques at 30 h post infection divided by bacterial CFU added per well. Error bars represent standard errors of the means from three independent experiments (*n* = 3 biological replicates). Asterisks indicate significant differences (*P* < 0.01, *t*-test).

We have previously demonstrated a role for *bimA* in the intercellular spreading of *B. pseudomallei* in HeLa cell monolayers using a plaque-forming efficiency assay (Sitthidet et al., 2011). Here, at 30 h post infection, HeLa cells infected with *B. pseudomallei* wild-type demonstrated numerous plaques within the cell monolayer. In contrast, no plaque formation was observed in cells infected with both Δ*bimC* and Δ*bimA* mutants. The Δ*bimC*/pBHR1-*bimC* complemented strain could partially restore this phenotype (Figure 3). Ablation of plaque formation is likely the result of the limited intracellular replication of the Δ*bimC* and Δ*bimA* mutants in HeLa cells, as shown in Figure 2B.

### BimC Is Required for *B. pseudomallei*–Induced MNGC Formation

**Figure 4:**
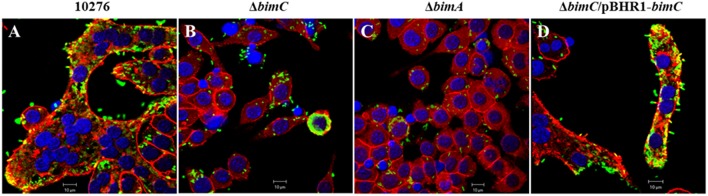
MNGC formation in J774A.1 cells. *B. pseudomallei* wild-type 10276 **(A)**, Δ*bimC* mutant **(B)**, Δ*bimA* mutant **(C)**, and Δ*bimC*/pBHR1-*bimC* **(D)** strains were used to infect J774A.1 cells. At 24 h post infection, the bacteria were stained with anti-*B. pseudomallei* lipopolysaccharide antibody and anti-rabbit-AlexaFluor^488^. F-actin (red) was stained with phalloidin-AlexaFluor^568^ and nuclei (blue) were stained with DAPI. Scale bar = 10 μm.

Another remarkable feature of *B. pseudomallei* infected host cells is the formation of multi-nucleated giant cells (MNGCs) in both phagocytic and non-phagocytic cell lines (Kespichayawattana et al., 2000). To assess whether *bimC* had an effect on *B. pseudomallei* induced MNGC formation, J774A.1 cells were infected with *B. pseudomallei*, Δ*bimC*, Δ*bimA*, and Δ*bimC*/pBHR1-*bimC* mutants and subjected to immunofluorescence staining. At 24 h post infection, MNGCs with 10–15 nuclei/cell were readily observed in cells infected with the wild-type strain (Figure 4A). In contrast, no MNGC formation was observed in cells infected with the Δ*bimC* mutant (Figure 4B) or the Δ*bimA* mutant (Figure 4C), despite the presence of mutant bacteria within the cytoplasm of these cells. Moreover, cells infected with the Δ*bimC*/pBHR1-*bimC* complemented strain displayed MNGC formation, albeit with fewer nuclei (3–5 nuclei/cell) (Figure 4D). The finding that cells infected with the Δ*bimC* and Δ*bimA* mutants failed to fuse despite harboring viable bacteria, may indicate a role for these proteins in the fusion process. Until recently the only bacterial factors implicated in this process where LfpA (Boddey et al., 2007), the T6SS-5 (Burtnick et al., 2011) and its associated effector protein Vgr-5 (Schwarz et al., 2014; Toesca et al., 2014).

### BimC Contributes to Pathogenesis of *B. pseudomallei* in a Surrogate *Galleria Mellonella* Model of Virulence

**Figure 5:**
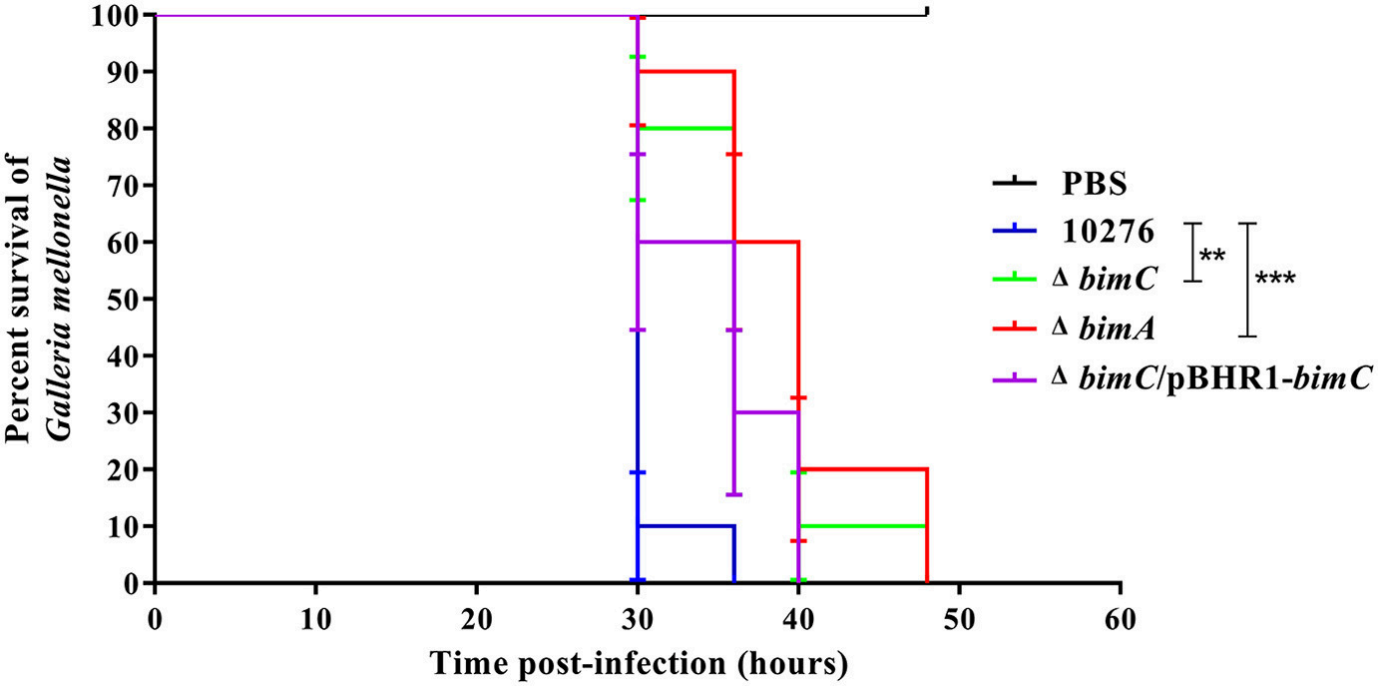
Virulence of *B. pseudomallei* strains in *Galleria mellonella* larvae. Representative data from an experiment where groups of 10 insect larvae were challenged with 100 CFU of either *B. pseudomallei* wild-type 10276, Δ*bimC* mutant, Δ*bimA* mutant, or the Δ*bimC*/pBHR1-*bimC* strain. The numbers of dead larvae were scored at 24, 30, 36, 40, and 48 h post infection. GraphPad Prism software was used to graph and analyze the data using a Log-rank (Mantel-Cox) test. Asterisks indicate significant differences (*P* < 0.05) in mean time to death (MTTD) between larvae infected with *B. pseudomallei* wild-type 10276 and the mutant strains. Data is representative of that obtained in three independent experiments (*n* = 3 biological replicates).

Data from the *in vitro* cell-based assays described above indicated that the BimC protein plays roles in various stages of the intracellular life of *B. pseudomallei* in both phagocytic and non-phagocytic cells. We next investigated the effect of *bimC* mutation on *B. pseudomallei* virulence in a *Galleria mellonella* larvae model. This model has been successfully employed to measure virulence of a wide range of bacterial pathogens including *Burkholderia* species (Wand et al., 2011). On three separate occasions, *G. mellonella* larvae were challenged with 100 CFU of *B. pseudomallei* strains (wild-type, Δ*bimC*, Δ*bimA*, and Δ*bimC*/pBHR1-*bimC*) and monitored for signs of melanization and death over a 48 h time period. Figure 5 shows representative data from a single experiment. In all experiments, infection of *Galleria* with either the Δ*bimC* or Δ*bimA* mutant showed attenuation of virulence demonstrated by a statistically significant mean time to death (MTTD, *p* < 0.05). In this assay system we were able to restore the virulence of the Δ*bimC* strain to levels similar to wild-type *B. pseudomallei* by expressing *bimC* on a plasmid (Δ*bimC*/pBHR1-*bimC*). This is the first demonstration of a role for the *bimC* gene in *B. pseudomallei* virulence.

## Discussion

Lu et al. (2014) have recently characterized the *B. thailandensis* BimC protein, demonstrating a role for this protein in actin-based motility and in the polar localization of BimA, using exogenously expressed tagged proteins. However, the role of *B. thailandensis* BimC in intracellular survival or pathogenesis was not assessed.

Here we have studied the importance of endogenously expressed *B. pseudomallei* BimC in actin-based motility, and confirm that it is absolutely required for this process. Furthermore, the *B. pseudomallei* BimC protein does not affect polar localization of native BimA, as demonstrated by the polar localization of BimA on the surface of Δ*bimC* bacteria in infected cells (Figure 1F and Figure S4F). This is contradictory to the findings of Lu et al. (2014), where exogenously expressed and tagged *B. thailandensis* BimC was required for the polar localization of the a similarly exogenously expressed and tagged *B. thailandensis* BimA protein. The presence of a 13 amino acid C-terminal extension on all of the available *B. pseudomallei* BimC protein sequences could account for this difference in function. Polar localization of the *B. pseudomallei* BimA protein may involve an alternative bacterial factor, or factors. Alternatively, it is possible that the study of an exogenously expressed *B. thailandensis* BimA protein containing a 3X HA tag between the extended signal sequence and the passenger domain, which are required for transport across the inner membrane via the Sec pathway and interaction with the Bam complex for transport across the outer membrane, respectively, may have abrogated its natural export route.

With regard to the role of BimC in intracellular life of *B. pseudomallei*, here we demonstrate a role for BimC in intracellular survival in HeLa cells. Ablation of the *bimC* gene resulted in a significant reduction in the net intracellular replication in HeLa cells (around 10-fold compared to wild-type bacteria at 24 h post infection) (Figure 2B), which also led to a complete lack of plaque formation in a cell monolayer at 30 h post infection (Figure 3). This data was mirrored by the Δ*bimA* mutant, which taken together suggests a key role for both of these proteins involved in actin-based motility in intracellular survival, cell to cell spread, and cell fusion events in this cell line. Perhaps of most interest is the finding that the requirement for both BimC and BimA in intracellular survival was not replicated in the murine macrophage-like J774.1 cells (Figure S5), although a defect in cell to cell spread was observed in this cell type (Figure 4). This may indicate a difference between the cell types in their innate immune responses to invading cytoplasmic bacteria. It is also possible that the macrophage-like cells require the presence of a priming agent such as LPS or IFN-γ for optimal bactericidal activity.

Finally, we utilized the surrogate *Galleria mellonella* larvae model of *B. pseudomallei* virulence to assess the role of both BimA and BimC in pathogenesis. A *bimA* insertion mutant has previously been shown to be attenuated following intraperitoneal injection of BALB/c mice (Lazar Adler et al., 2015) and intranasal infection of C57/Bl mice (M. Stevens and G. Bancroft, unpublished data), a finding that we reproduced in the *G. mellonella* model using a *bimA* deletion mutant. In addition, significant attenuation of the Δ*bimC* mutant was also observed, which was restored by expression of *bimC in trans*, confirming a role for BimC in virulence.

Whilst we have demonstrated a role for *B. pseudomallei* BimC in intracellular survival in epithelial cells, BimA-mediated actin-based motility and virulence, we do not know how this protein affects these processes directly. Using a Yeast two hybrid approach, Lu et al. (2014) showed that the *B. thailandensis* BimC protein is an iron-binding protein that directly interacts with the *B. thailandensis* BimA protein. The authors also showed that the *B. thailandensis* BimC protein lacks the predicted glycosyltransferase activity displayed by other members of the BAHT family of glycosyltransferases (Lu et al., 2014), and were unable to hypothesize an alternative biochemical function for the *B. thailandensis* BimC protein. Since the *B. pseudomallei* BimC protein clearly does not play a role in the polar localization of the cognate BimA protein, and is unlikely to be a functional glycosyltransferase; further molecular studies will be required to unravel the mechanism underlying the role of the *B. pseudomallei* BimC protein in pathogenesis of this fascinating micro-organism.

## Conclusion

Here we have studied the importance of the BimC protein of *B. pseudomallei*, the causative agent of melioidosis, in the intracellular life of the pathogen. We have shown that the protein is required for actin-based motility within the host cell cytoplasm. This is associated with a role in intracellular survival and cell to cell spread of the bacterium in HeLa cells, and a significant role in virulence in a surrogate insect model system.

## Author Contributions

VS carried out the experiments and was involved in the preparation of the manuscript. SC carried out the experiments. SK was involved in the study design and manuscript preparation. JS contributed to the study design, experiments, and manuscript writing.

### Conflict of Interest Statement

The authors declare that the research was conducted in the absence of any commercial or financial relationships that could be construed as a potential conflict of interest.
