# Supplementary material for: Burkholderia pseudomallei BimC Is Required for Actin-Based Motility, Intracellular Survival, and Virulence
Source: Front Cell Infect Microbiol. 2019 Mar 22;9:63. doi: 10.3389/fcimb.2019.00063 (PMC6439308; doi:10.3389/fcimb.2019.00063)

**SUPPLEMENTARY FIGURES**

**FIGURE S1 Analysis of BimC C-terminal amino acid sequences.** (A) Clustal Omega multiple sequence alignment of C-terminal amino acids of BimCs encoded by multiple *B. pseudomallei*, *B. mallei*, *B. thailandensis* and *B. oklahomensis* strains. *B. pseudomallei* sequences are shown in green, *B. mallei* in red, *B. thailandensis* in blue and *B. oklahomensis* in purple. Two of the conserved cysteines involved in iron-binding of the *B. thailandensis* BimC protein are highlighted by red asterisks on the bottom row. Black asterisks indicate fully conserved residues, : and . indicate replacement of residues with other amino acids with highly and weakly similar properties respectively, on the bottom row of the table. A lack of a symbol in this row indicates a complete lack of conservation at this position. (B) Phylogenetic tree constructed in Clustal W2-phylogeny.

**FIGURE S2 Construction of the *B. pseudomallei*** Δ***bimC* deletion mutant.** (A) Gene organization of the *bim* locus on *B. pseudomallei* chromosome 2. Black arrows indicate the intended region of *bimC* deletion. (B) Verification of the putative *B. pseudomallei* Δ*bimC* deletion mutant by PCR using primers flanking the deletion site. The DNA amplicons from *B. pseudomallei* wild-type (lane 2), *bimC* merodiploid (lane 3) and Δ*bimC* deletion mutant using primers flanking the *bimC* gene are shown. Lane 1: DNA ladder.

**Figure S3. Co-localization of *B. pseudomallei* strains with the phagosomal marker protein LAMP-1 in infected J774A.1 cells.** (A) Representative confocal micrographs showing the association of intracellular *B. pseudomallei* wild-type strain 10276, Δ*bimC*, Δ*bimA* mutant and T3SS deficient *bsaZ*::pDM4 mutants with LAMP-1 in J774A.1 cells. Bacteria (red) were stained with mouse anti-*B. pseudomallei* monoclonal antibody (9D5) and detected with anti-mouse antibody-AlexaFluor^594^. Host cell LAMP-1 (green) was stained with rat monoclonal antibody (1D4B) and detected with anti-rat antibody-AlexaFluor^488^. Nuclei (blue) were stained with DAPI. (B) Quantitative analysis of *B. pseudomallei* strain co-localization with LAMP-1 in J774A.1 infected cells. The percentage of co-localization was calculated by the number of bacteria co-localized with LAMP-1 x 100/total number of intracellular bacteria. Error bars represent standard error of the mean for data collected from three independent experiments (n=3 biological replicates). Asterisks indicate significant differences (*P*<0.01*,* students *t*-test) between *B. pseudomallei* wild-type 10276 and mutant strains. Scale bar = 10μm.

**FIGURE S4 Actin-based motility of *B. pseudomallei* in infected J774A.1 cells.** *B. pseudomallei* wild-type 10276 (A and E), Δ*bimC* mutant (B and F), Δ*bimA* mutant (C and G) and Δ*bimC*/pBHR1-*bimC* (D and H) strains were used to infect J774A.1 cells. At 12 hours post infection, the infected macrophages were stained for bacteria and actin-tails (A-D), or bacteria and BimA protein (E-G). Bacteria (green) were stained using mouse monoclonal anti-*B. pseudomallei* lipopolysaccharide antibody and detected with anti-rabbit antibody-AlexaFluor^488^. F-actin (red) was stained with phalloidin-AlexaFluor^568^ and nuclei (blue) were stained with DAPI. BimA protein (red) was stained with a panel of three monoclonal antibodies detected with anti-mouse antibody-AlexaFluor^568^. Scale bar = 5μm.

**FIGURE S5 Intracellular replication and survival of *B. pseudomallei* strains within J774A.1 murine macrophage-like cells.** J774A.1 cells were infected with *B. pseudomallei* wild-type strain 10276 (black bars), Δ*bimC* mutant (white bars), Δ*bimA* mutant (dotted bars) or Δ*bimC*/ pBHR1-*bimC* (gray bars) strains. At the indicated time point, the numbers of viable intracellular bacteria (CFU) were determined. Error bars represent standard errors of the means from three independent experiments (n=3 biological replicates).

**Supplementary methods**

**Confocal analysis of bacterial co-localization with LAMP-1**

To examine the intracellular localization of *B. pseudomallei* wild-type and mutant strains in J774A.1 cells relative to lysosome associated membrane protein 1 (LAMP-1) containing vesicles, cells were infected and fixed two hours post-infection. As a control for this experiment, we included a previously characterized strain with mutation in a gene encoding a major structural protein of the Bsa Type Three Secretion System, which has been proven to be retained within the phagocytic compartment of infected cells and demonstrate a significant delay in escape into the cytoplasm (*bsaZ*::pDM4). After fixation, permeabilization and blocking steps, the bacteria were stained red with mouse anti-*Burkholderia* monoclonal antibody (9D5; a kind gift from Dr. Narissara Chantratita, Faculty of Tropical Medicine, Mahidol University) and Alexa Fluor 594-goat anti-mouse IgG antibody (Invitrogen). LAMP1 was stained green with rat monoclonal antibody (1D4B; Abcam, Cambridge, UK) and Alexa Fluor 488-goat anti-rat IgG antibody. Nuclei were stained blue with DAPI. The infected cells were analyzed by confocal laser scanning microscopy using a Zeiss LSM 510 META instrument (Carl Zeiss, Germany). Bacteria were considered to be associated with LAMP-1 if any region of the red fluorescent stained bacterial cells co-localized with the green fluorescent stained LAMP-1-positive vacuoles, visualized in yellow in the merged image. The percentage of intracellular *Burkholderia* associated with LAMP-1 was determined as the number of bacteria co-localized with LAMP-1/total number of intracellular bacteria x100. For the quantitative analysis of the association of intracellular *B. pseudomallei* strains with LAMP-1 containing vesicles, at least 200 individual bacteria were monitored.

**Table S1 Oligonucleotides used in this study**


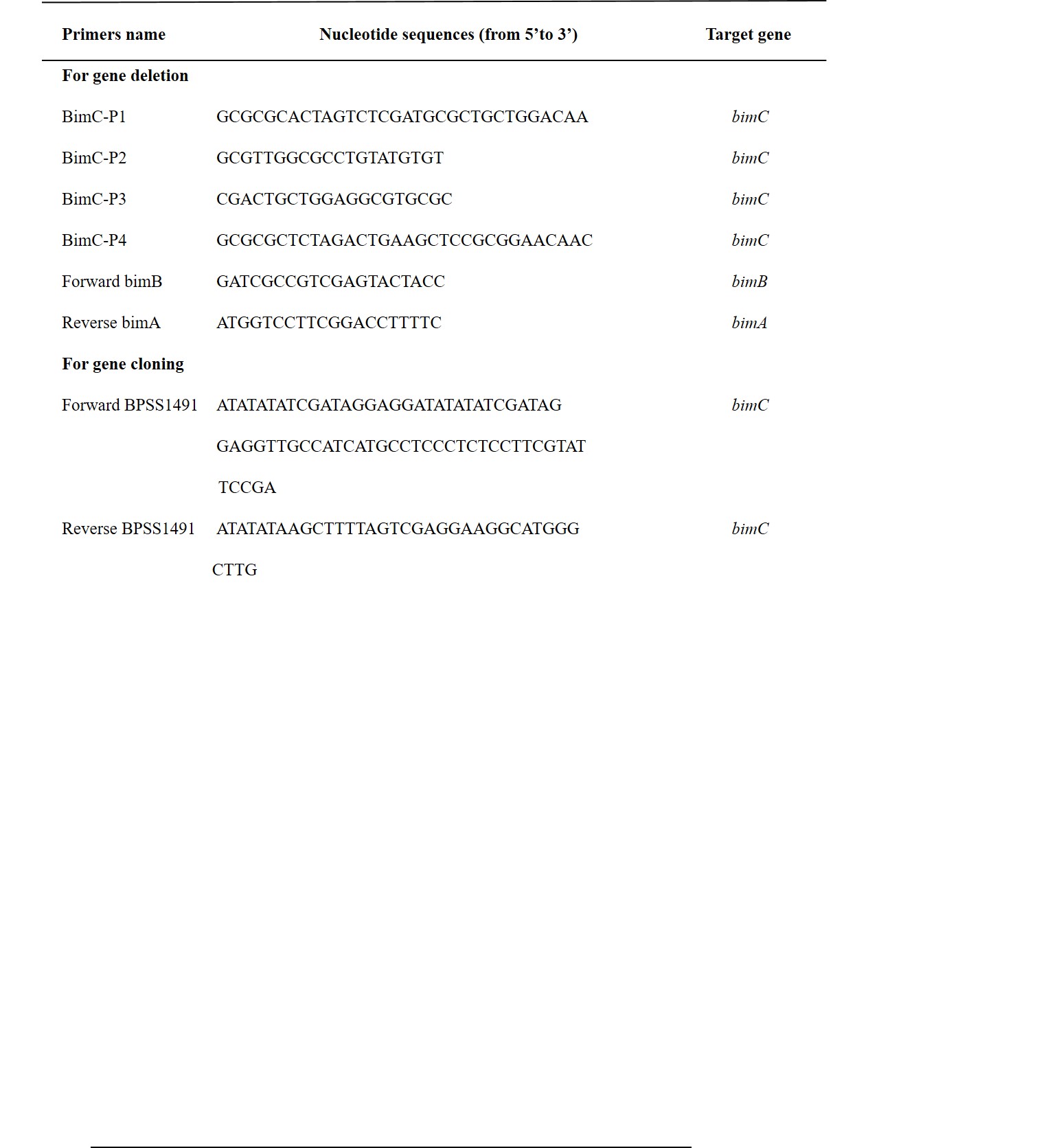

Supplement: Supplementary file 1 [file Data_Sheet_1.docx]
