# Supplementary figures and images for: Burkholderia pseudomallei BimC Is Required for Actin-Based Motility, Intracellular Survival, and Virulence
Source: Front Cell Infect Microbiol. 2019 Mar 22;9:63. doi: 10.3389/fcimb.2019.00063 (PMC6439308; doi:10.3389/fcimb.2019.00063)

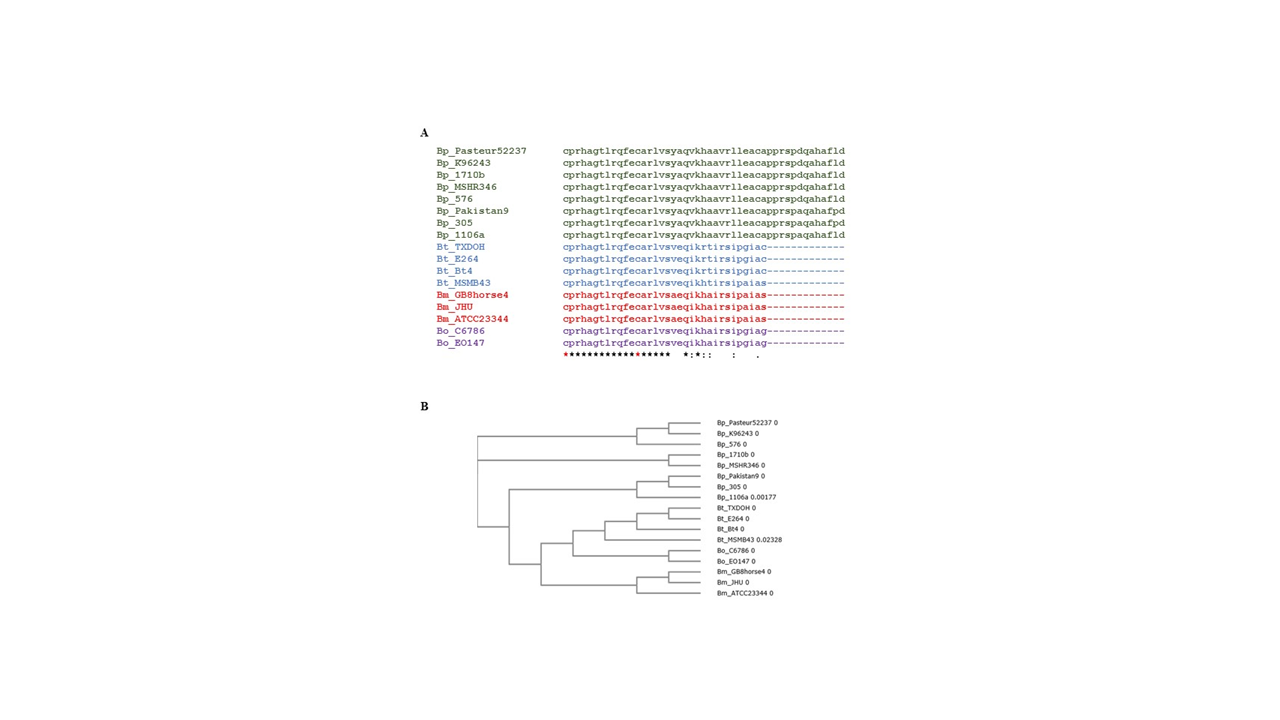

Supplement: Supplementary file 2 [file Image_1.tif]

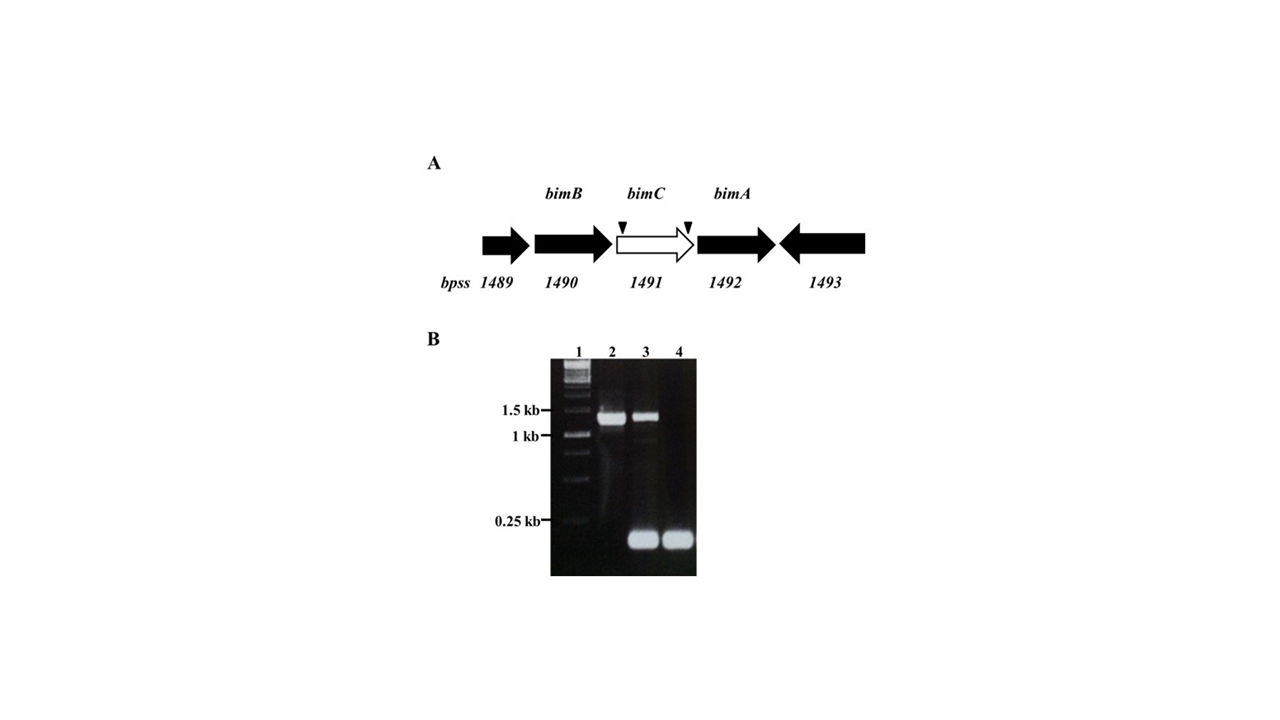

Supplement: Supplementary file 3 [file Image_2.tif]

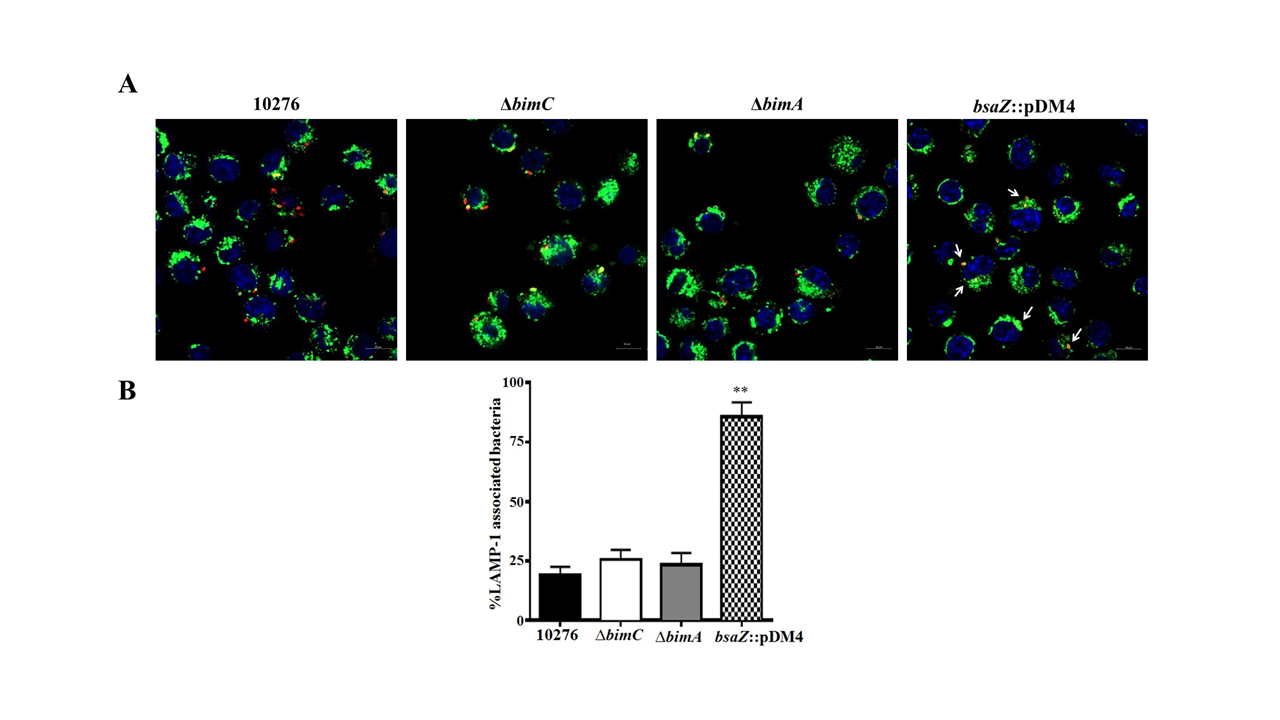

Supplement: Supplementary file 4 [file Image_3.tif]

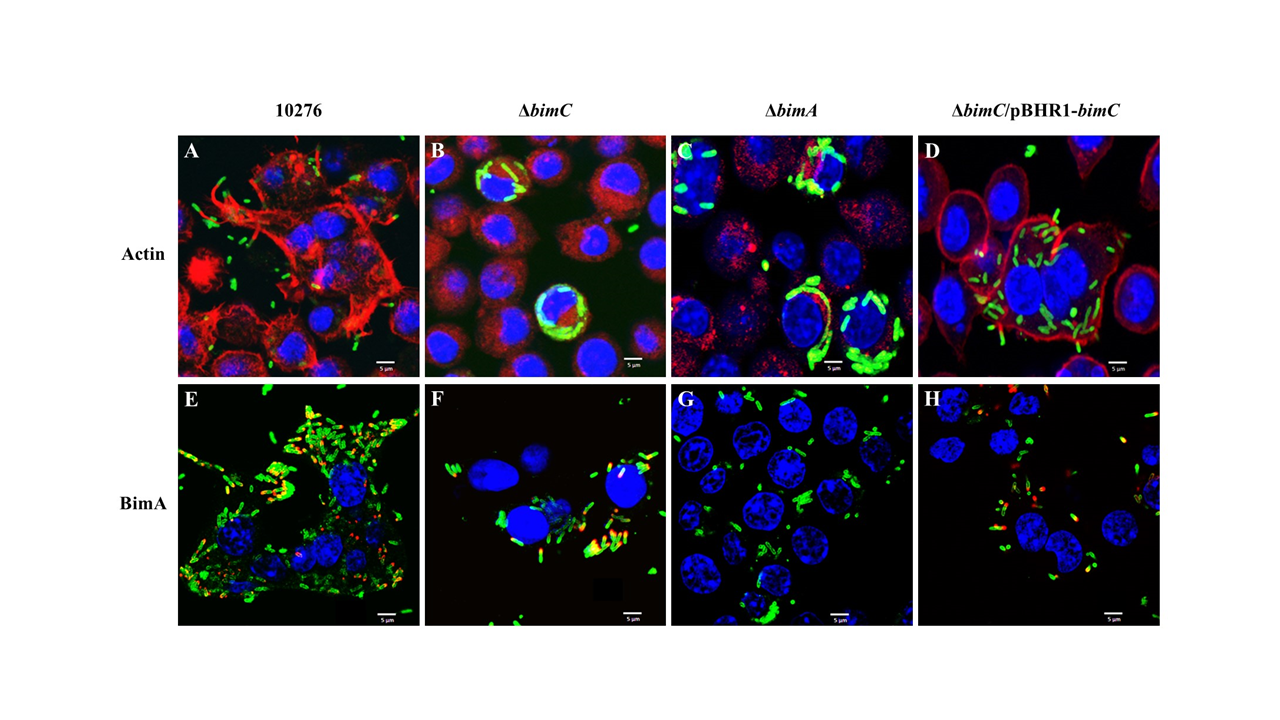

Supplement: Supplementary file 5 [file Image_4.tif]

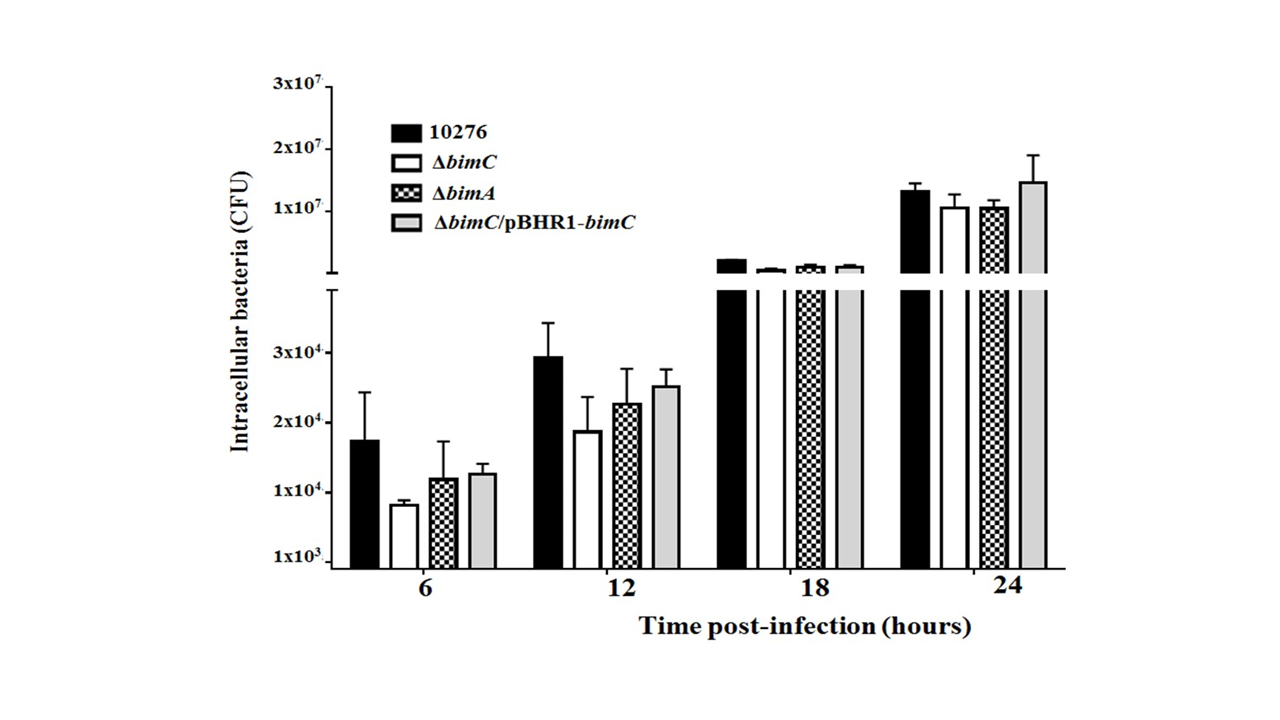

Supplement: Supplementary file 6 [file Image_5.tif]
